# It is time to change the definition: Endometriosis is no longer a pelvic disease

**DOI:** 10.1016/j.clinsp.2024.100326

**Published:** 2024-02-06

**Authors:** Maria Carolina Machado da Silva, Luiz Philipe de Souza Ferreira, Amanda Della Giustina

**Affiliations:** a Neuropharmacology Laboratory, Department of Pharmacology, Universidade Federal de Minas Gerais, Belo Horizonte, MG, Brazil; b Department of Morphology and Genetics, Structural and Functional Biology Graduate Program, Escola Paulista de Medicina, Universidade Federal de São Paulo, São Paulo, SP, Brazil; c Sprott Centre for Stem Cell Research, Ottawa Hospital Research Institute, Ottawa, ON, Canada

Endometriosis is a chronic, inflammatory, hormone-dependent gynecologic condition characterized by the presence of endometrial-like tissue outside the uterine cavity,^1^ including pelvic peritoneal surfaces, rectovaginal space, ligaments, ovaries, as well as the bowel and bladder.^2^ The presentation of endometriosis is highly variable, ranging from superficial peritoneal lesions of different colors to ovarian cysts (endometrioma), deep infiltrating nodules, and extra pelvic lesions.^3^ In relation to symptoms, women with endometriosis may experience cyclic or constant severe pelvic pain, which can also occur during and after sexual intercourse (dyspareunia), accompanied by dysuria, dyschesia, dysmenorrhea, and infertility, among others.^4^

Approximately 10 % of women of reproductive age worldwide are affected by this disease,^5^ although the exact prevalence remains uncertain due to factors such as the presence of asymptomatic individuals, difficulties in accessing healthcare, and in diagnosis.^6^ Studies show that women endure five to 12 years until getting diagnosed and adequate treatment,^7^, ^8^, ^9^, ^10^ and the long diagnostic delay leads to disease progression and more pre-diagnosis symptoms that increase the costs and the utilization of healthcare systems, and negatively impact the quality of life.^11^^,^^12^ Furthermore, the current alternatives for endometriosis treatment rely on controlling the symptoms either by medication – mostly, hormonal-based options that may cause a wide range of side effects – or surgery, subjecting the affected women to circumstances where life-changing decisions must be made based on a restricted array of possibilities to avoid disease worsening or recurrence, to preserve fertility, and diminish pain.^13^

In addition, women with this condition frequently experience endometriosis-related stigma (endo-stigma), in which the individuals are devaluated or discredited, and have their symptoms and the social impact of this condition dismissed by either society or the healthcare community.^14^ A qualitative study pointed out that menstrual pain is seen as a normal part of women's lives by family members of Latina women living with endometriosis, and that these women often feel invalidated, refraining from sharing their symptomatology to avoid suffering.^15^ Moreover, the “etiquette of menstruation”,^16^ enforced in many countries as a cultural rule, contributes to the endo-stigma and lack of awareness about possible irregularities involving menstruation: women are discouraged to talk about their symptoms since menstruation is considered a private matter and should not be openly discussed, especially with men. Noteworthy, it is common that other women, such as mothers and friends, encourage this concealment and perpetuate the stigma by normalizing other's painful symptoms, based on the understanding that women who speak about their menstruation could be ostracised, criticized, and considered weak.^17^^,^^18^ Importantly, anticipating stigma and the lack of social support are important stressors that impair mental well-being, and the consequent disruption in social, educational, and professional obligations caused by living with endometriosis is an extra load of psychological burden.^19^

A growing body of evidence indicates that several endometriosis-related aspects play a negative role in the quality of life of those affected. Fonseca et al. evaluated the relationships among various pain symptoms due to endometriosis and they identified chronic pelvic pain and dysmenorrhea as the most important pain symptoms impacting the women's quality of life.^20^ On the other hand, another study observed that the reduction in quality of life was dependent on the types of dyspareunia, irrespective of the disease severity.^21^ An international cross-sectional survey found that chronic pelvic pain, dyspareunia, and the number of co-morbidities were independent factors in a regression analysis that significantly reduced both the physical and mental aspects of the quality of life of the participants.^22^ However, apart from the experienced physical pain, uncertainty is another important factor that negatively influences the quality of life: women with endometriosis often sense uncertainty about diagnosis, disease progression, and how it will impact their future, e.g., the possibility of disease relapse after surgery, premature menopause, and fertility issues.^23^^,^^24^

Taken together, these factors contribute to the burden of endometriosis and may favor the relationship between the disease and the development of mental illnesses, such as anxiety and mood disorders.^25^ Among a small sample of 24 adolescents and young women diagnosed with endometriosis, González-Echevarría and colleagues demonstrated that 45.8 % of the participants had moderate-severe levels of anxiety, and 33.4 % showed moderate-severe depressive levels.^26^ Similar findings were noted in Hungarian women: over 54 % of the individuals presented with anxiety and 20 % with depressive symptoms.^27^ In a prospective cross-sectional study, Lorencatto et al. observed that women with chronic pain caused by endometriosis presented a higher prevalence of depression when comparing those to patients without pain.^28^ The influence of endometriosis on mental health aspects was also seen in Puerto Rican women: approximately 50 % of the participants felt depressed due to endometriosis-related fertility issues, and almost 40 % of them declared concerns about the sexual aspects of personal relationships and inefficacy of treatments.^29^ An alarmingly higher prevalence of depression was found in a group of 205 Romanian women diagnosed with endometriosis-caused infertility: 98.5 % of the participants showed severe depression because of the endometriosis influence on fertility outcomes.^30^

Even though the healthcare accessibility and the implications of existing treatment options, as well as social determinants and stigma, may interfere with mental aspects in these women and thus must be taken into consideration, the involvement of mental illnesses in endometriosis should also be attributed to central pathophysiological changes. The hypothesis of a brain–body–brain cross-talk”, introduced by Tariverdian and colleagues,^31^ points out that the peripheral alterations provoked by endometriosis, such as peritoneal inflammation and angiogenesis, would have central repercussions since inflammatory mediators might enter the Central Nervous System (CNS) bypassing/crossing the Blood-Brain Barrier (BBB) or via stimulation of vagal nervous afferents. Also, clinical symptoms, like pain and infertility, would favor a high-stress perception, which would be aggravated by the sickness response caused by the central inflammatory state. Consequently, the peripheral alterations and the clinical symptoms undergo positive feedback by the release of catecholamines, growth factors, peptides, and stress-related hormones, thus perpetuating the effects of endometriosis in the CNS. Recently, the approach proposed by Appleyard et al. places stress, regardless of type, as a central mechanism exacerbating endometriosis by triggering the Hypothalamic-Pituitary-Adrenocortical (HPA) axis and its ultimate dysregulation due to chronic inflammation.^32^ A similar theory, proposed by Ghosh et al., poses endometriosis-linked stress as a key factor influencing the desynchronization of both the HPA and the Hypothalamic-Pituitary-Gonadal (HPG) axis, as well as the circadian system as a response to inflammatory stimuli.^33^

Corroborating these theories, experimental studies have demonstrated that endometriosis can affect the CNS in different ways. Mice with endometriosis were found to have behavior suggestive of depression and anxiety, as well as increased pain sensitivity, and this was accompanied by alteration in gene expression and brain electrophysiology in brain regions involved in pain perception, mood, and anxiety disorders.^34^ Also, endometriosis increased the expression of reactivity markers, such as GFAP and IBA-1, and promoted morphological changes in glial cells in the spinal cord horn^35^^,^^36^ and various brain regions, including the hippocampus and the hypothalamus.^37^ Moreover, Samani and colleagues showed that endometriosis-derived cells can migrate and engraft to distant organs outside the pelvic cavity, including the brain,^38^ indicating that endometriosis lesions could develop in the CNS. Supporting this statement, a recent case report and literature review publication depicts several cases of cerebral endometriosis and details the case of a patient who developed psychiatric disorders due to endometriosis lesions in the brain.^39^

In conclusion, it is time for the healthcare and scientific communities to recognize endometriosis no longer as a pelvic, but rather a systemic condition. This disease represents an enormous burden on the lives of those affected, impacting their quality of life and mental well-being due to its pathophysiology and its influence on psychosocial aspects. It also constitutes a challenge to the healthcare system due to its elevated public health costs, insufficient knowledge about the disease which contributes to delayed diagnosis, and the reduced availability of treatments. Thus, it is urgent the necessity of creating awareness about this condition to improve the quality of life of those affected and to provide more adequate treatment options that consider the systemic effects of endometriosis.

## Conflicts of interest

The authors declare no conflicts of interest.
